# Molecular Epidemiology of Rabies in Southern People’s Republic of China

**DOI:** 10.3201/eid1508.081551

**Published:** 2009-08

**Authors:** Xiao-Yan Tao, Qing Tang, Hao Li, Zhao-Jun Mo, Hong Zhang, Ding-Ming Wang, Qiang Zhang, Miao Song, Andres Velasco-Villa, Xianfu Wu, Charles E. Rupprecht, Guo-Dong Liang

**Affiliations:** Chinese Center for Disease Control and Prevention, Beijing, People’s Republic of China (X.-Y. Tao, Q. Tang, H. Li, Q. Zhang, M. Song, G.-D. Liang); Guangxi Center for Disease Control and Prevention, Nanning, People’s Republic of China (Z.-J. Mo); Hunan Center for Disease Control and Prevention, Changsha, People’s Republic of China (H. Zhang); Guizhou Center for Disease Control and Prevention, Guiyang, People’s Republic of China (D.-M. Wang); Centers for Disease Control and Prevention, Atlanta, Georgia, USA (A. Velasco-Villa, X. Wu, C.E. Rupprecht)

**Keywords:** Molecular epidemiology, nucleoprotein, rabies, epidemic, China, viruses, research

## Abstract

In recent years, the number of human rabies cases in the People’s Republic of China has increased during severe epidemics in 3 southern provinces (Guizhou, Guangxi, and Hunan). To analyze the causes of the high incidence of human rabies in this region, during 2005–2007, we collected 2,887 brain specimens from apparently healthy domestic dogs used for meat consumption in restaurants, 4 specimens from suspected rabid dogs, and 3 from humans with rabies in the 3 provinces. Partial nucleoprotein gene sequences were obtained from rabies-positive specimens. Phylogenetic relationships and distribution of viruses were determined. We infer that the spread of rabies viruses from high-incidence regions, particularly by long-distance movement or transprovincial translocation of dogs caused by human-related activities, may be 1 cause of the recent massive human rabies epidemics in southern China.

## Abstract 2

Migration and transport of dogs may have caused recent epidemics of human rabies.

Rabies has been endemic to the People’s Republic of China for many years; domestic dogs act as the main reservoir (*1*–*3*). After efforts to stabilize the dog population, the lowest incidence of human rabies (159 cases) was recorded in 1996. Subsequent annual increases followed: 505 cases were reported in 2000, 1,191 cases in 2002, and 2,651 cases in 2004 (*2*,*4*). In 2005, three southern provinces (Guizhou, Guangxi, and Hunan) recorded most of the human rabies cases throughout China (*5*). During the next 2 years, this region had ≈50% (6,595) of the total number of cases in China and contributed to one of the most serious human rabies epidemics in this country (*5*,*6*).

Various studies have been conducted to determine the causes and characteristics of rabies epidemics in China. Descriptive epidemiologic analysis showed that the increase in domestic dog populations and low vaccination coverage have contributed to rabies epidemics in the 3 provinces in southern China (*7*–*9*). Other authors have suggested that this trend may be caused by a carrier state in healthy dogs that remains undetected (*10*). Molecular epidemiologic analysis of dog specimens collected from 5 provinces (Guangxi, Hunan, Guizhou, Jiangsu, and Henan) demonstrated that rabies viruses (RABVs) in China are similar to those reported from previous epidemics (*11*). However, no studies have investigated the phylogenetic relationships among viruses circulating in different provinces during peaks of rabies incidence.

RABV is the prototype member of the family *Rhabdoviridae* and the genus *Lyssavirus*. It encodes 5 structural proteins: nucleoprotein, phosphoprotein, matrix protein, glycoprotein, and RNA-dependent RNA polymerase (*12*). The nucleoprotein (N) gene has been extensively used for genetic typing and evolutionary studies because of its relatively conserved variation among reservoir-associated variants and geographic lineages (*13*–*16*). Since the onset of a human rabies epidemic in China in 2005, we have collected brain specimens from dogs and human patients. Partial N gene sequences were obtained in an attempt to understand the sustained increase in rabies cases in China by analysis of RABVs circulating in the 3 southern provinces with the highest rabies incidence.

## Methods

### Specimen Collection

**Figure 1:**
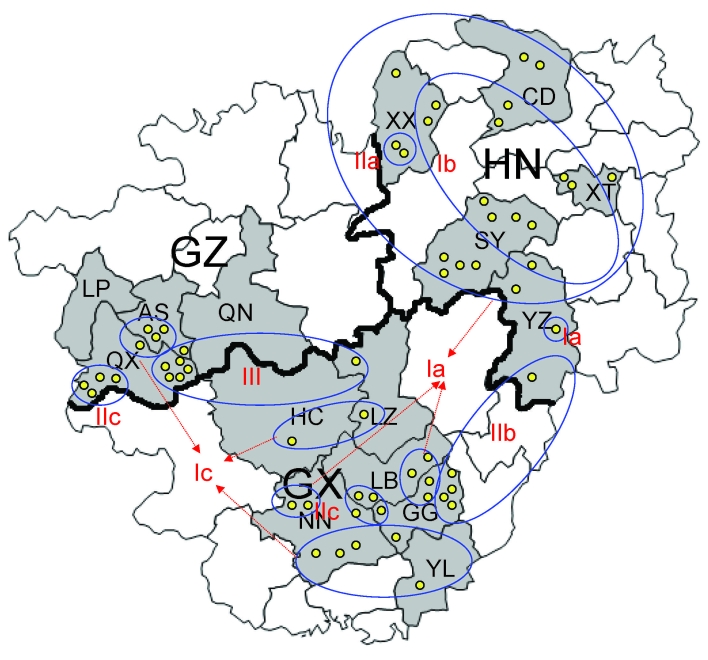
Locations of 15 cities selected for specimen collection in Guizhou (GZ), Hunan (HN), and Guangxi (GX) provinces, in southern People’s Republic of China, 2005–2007, and genetic groups and subgroups of 60 samples analyzed for rabies virus. Roman numerals and letters indicate genotypes, gray areas indicate regions selected for specimen collection, yellow circles indicate specimens collected, ovals indicate regions with the same genotype, and arrows indicate specimens with the same genotype. LP, Liupanshiu; AS, Anshun; QN, Qiannan; QX, Qianxinan; XX, Xiangxi; CD, Changde; XT, Xiangtan; SY, Shaoyang; YZ, Yongzhou; HC, Hechi; LZ, Liuzhou; LB, Laibin; GG, Guigang; NN, Nanning; YL, Yulin.

From 2005 through 2007, we randomly collected 2,887 brain specimens from apparently healthy domestic dogs used as meat in restaurants of 15 cities in Guizhou, Guangxi, and Hunan provinces (Table 1, Figure 1). In addition, we included 4 brain specimens of suspected rabid dogs that had bitten other animals or humans and 3 brain specimens from patients who had died of rabies.

**Table 1:** Diagnostic results for rabies virus in southern People’s Republic of China, 2005–2007*

Location	No. specimens	No. (%) positive
Guizhou Province		
Qianxinan	427	13 (3)
Qiannan	54	1 (1.9)
Anshun	173	8 (4.6)
Liupanshui	200	0
Guangxi Province		
Laibin	213	4 (1.9)
Guigang	304	8 (2.6)
Hechi	206	1 (0.4)
Yulin	302	4 (1.3)
Nanning	222	7 (3.2)
Liuzhou	105	2 (1.9)
Hunan Province		
Shaoyang	151	6 (4)
Yongzhou	162	2 (1.2)
Xiangtan	114	3 (2.6)
Changde	102	4 (3.9)
Xiangxi	152	3 (1.9)
Total	2,887	66 (2.3)

*Samples were obtained from apparently healthy domestic dogs.

### Detection and Sequencing of RABV

All specimens were examined by using a direct immunofluorescence assay (DFA) with fluorescent-labeled monoclonal antibody against RABV N protein (Rabies DFA Reagent; Chemicon Europe Ltd., Chandlers Ford, UK). For all identified RABV specimens, RNA was extracted with TRIzol Reagent (Invitrogen, Carlsbad, CA, USA) and used as template for cDNA synthesis with Ready-To-Go You-Prime First-Strand Beads (Amersham Pharmacia Bioscience, Chalfont St. Giles, UK). The 720-nt sequence of the N gene, encoding regions corresponding to nt 704–nt 1423 of the total genetic sequence of the Pasteur strain of RABV, was amplified with primers N644F (5′-AAGATGTGYGCYAAYTGGAG-3′, nt 644–nt 663) and N1537R (5′-GGATTGACRAAGATCTTGCTCAT-3′, nt 1515–nt 1537). Locations of primer sequences were referred to the full genome sequence of the Pasteur strain of RABV (M13215). PCR products were purified by using the QIAquick PCR Purification Kit (QIAGEN Ltd., Crawley, UK) and sequenced with an ABI PRISM 3100 DNA sequencer (Applied Biosystems, Foster City, CA, USA).

### Sequence Analysis

Complete alignment of nucleotide sequences was performed by using the ClustalX program, version 1.8 (*17*). MegAlign software version 5 (DNAStar, Inc., Madison, WI, USA) was used to analyze nucleotide and deduced amino acid sequence homologies. The neighbor-joining method in MEGA3 version 3.1 (*18*) was used with 1,000 bootstrap replications (*19*). All taxa used for the comparative analyses were obtained from GenBank (Table 2).

**Table 2:** Lyssavirus strains used in study of molecular epidemiology of rabies virus in southern People’s Republic of China, 2005–2007

Genotype	Strain	Year	Host species or type of strain	Origin	GenBank accession no.
GT1	SC02–90	2002	Dog	Indonesia	AB154243
	D664_45	–	Dog	Thailand	DQ267925
	CTN	1956	Vaccine	China	AF367863
	483a	–	Arctic fox	Russia	AY352487
	Ontario type 2/4	1991	Fox	Canada	U11734
	–	–	–	South Africa	AF467949
	FluryLEP	1939	Vaccine	USA	DQ099524
	PM1503	–	Vaccine	–	DQ099525
	SRV9	–	Vaccine	China	AF499686
	SAD B19	1935	Vaccine	USA	M31046
	CVS	–	Laboratory strain	USA	AF406696
	ERA	1935	Vaccine	USA	AF406695
	PV	1965	Vaccine	France	M13215
	92RABL00867	–	Skunk	Canada	AF344306
	3789	1998	Skunk	USA	AF461045
	Eth2003	–	Wolf	Ethiopia	AY500827
	RC-HL	–	Vaccine	Japan	D16331
	3aG	1931	Vaccine	China	AF155039
	DRV	2002	–	China	DQ875051
	ISR-40	2000	Fox	Israel	DQ837421
	–	–	Fox	Europe	AF045664
	3502f	–	Red fox	Russia	AY352455
	–	–	Dog	India	AF374721
	1294	1986	Dog	Sri Lanka	AY138549
	2054	2001	Bat	USA	AF394888
	V920	1993	Bat	Mexico	AY877435
	BR-BAT2	–	Bat	Brazil	AB201820
GT7	Ballina	1996	Bat	Australia	AF006497
GT5	8615POL	1985	Bat	Poland	U22844
GT4	86132AS	1986	Human	South Africa	U22848
GT6	9007FIN	1986	Human	Finland	U22846
GT3	MOK	1981	Cat	Zimbabwe	U22843
GT2	8619NGA	1958	Bat	Nigeria	U22842

## Results

### Specimen Detection

Of 2,887 specimens randomly collected from dogs, 66 were positive for RABV (positivity rate = 2.3%) (Table 1). The 7 additional specimens obtained from 4 dogs suspected of having rabies and 3 patients who died of rabies all were positive for RABV.

### Sequencing

A 720-bp region of the N gene of the RABV-positive specimens was obtained from 60 specimens (53 sequences were from 66 specimens obtained from healthy dogs and 7 sequences were from the 4 rabid dogs and 3 patients who died of rabies). The 13 other positive specimens were not sequenced. All 60 sequences were submitted to GenBank (accession nos. EF990564–EF990623).

### Similarity Analysis

A sequence comparison of 60 N gene fragments showed 87.6%–100% nucleotide similarity. Some specimens from the same province were identical in their entire nucleotide sequence: 6 specimens from Guangxi Province (CGX0602D, CGX0604D, CGX0605D, CGX0513D, CGX0620D, and CGX0622D); 3 specimens from Hunan Province (CHN0505D, CHN0506D, and CHN0507D); and 2 specimens from Guizhou Province (CGZ0506D and CGZ0514D). Identical sequences were also observed in different provinces: 7 specimens from Hunan Province (CHN0614D, CHN0516D, CHN0529D, CHN0609D, CHN0615D, CHN0528D, and CHN0525D) and 2 specimens from Guizhou Province (CGX0612D and CGX0606D), 2 specimens from Guizhou Province (CGZ0512D and CGZ0516D) and 3 specimens from Guangxi Province (CGX0614D, CGX0523D, and CGX0625D), and 4 specimens from Guizhou Province (CGZ0501D, CGZ0505D, CGZ0513D, and CGZ0518D) and 1 specimen from Guangxi Province (CGX0617D). Identity levels of deduced amino acid sequences of the 60 specimens ranged from 95.4% to 99.6%.

### Phylogenetic Analysis

**Figure 2:**
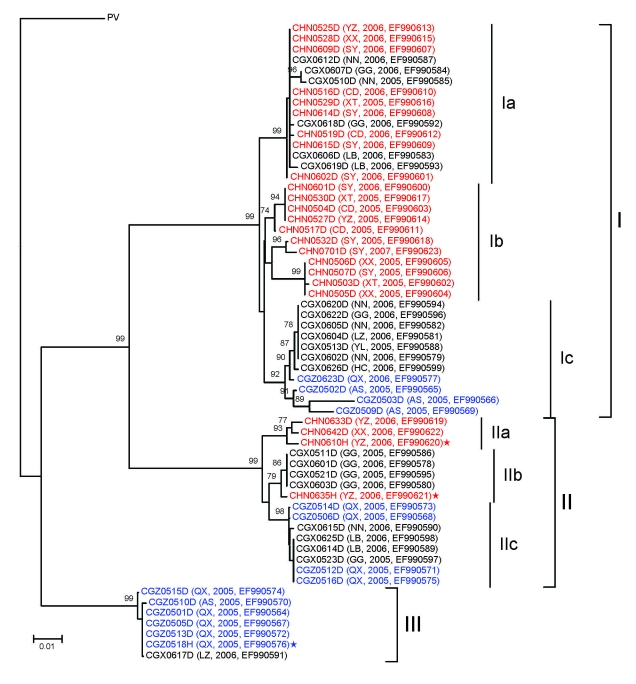
Neighbor-joining phylogenetic tree of 60 specimens of rabies virus from the People’s Republic of China, 2005–2007, based on a 720-nt (nt 704–nt 1423) nucleoprotein (N) gene fragment of rabies virus rooted with the Pasteur strain of rabies virus (PV). Numbers at each node indicate degree of bootstrap support; only those with support >70% are indicated. Taxa are from Hunan Province are shown in red, taxa are from Guangxi Province in black, and taxa are from Guizhou Province in blue. City, year of sample collection, and GenBank accession no. are included for each taxon. Stars indicate human samples; others are dog samples. YZ, Yongzhou; XX, Xiangxi; SY, Shaoyang; NN, Nanning; GG, Guigang; CD, Changde; XT, Xiangtan; LB, Laibin; LZ, Liuzhou; YL, Yulin; HC, Hechi; QX, Qianxinan; AS, Anshun. Scale bar indicates nucleotide substitutions per site.

Results of a phylogenetic analysis of 60 sequences obtained in this study are shown in Figure 2; the Pasteur strain of RABV was used as an outgroup. The 60 virus specimens clustered into 3 statistically supported branches (bootstrap support 99%) and were designated as groups I, II, and III. Groups I and II can be further divided into 3 subgroups. Subgroup Ia contained specimens from Hunan Province (n = 9) and Guangxi Province (n = 6). All specimens in subgroup Ib were from Hunan Province. Most (7/11) specimens in subgroup Ic were from Guangxi Province, with 4 from Guizhou Province. The 3 specimens in subgroup IIa were from Hunan Province. Six of 7 specimens in subgroup IIb were from Guangxi Province; the other specimen was from Hunan Province. Subgroup IIc contained 4 specimens from Guangxi Province and 4 from Guizhou Province. Most (6/7) specimens in group III were from Guizhou Province; the other specimen was from Guangxi Province (Figure 1).

Human virus specimens showed close relationships to dog virus specimens from the same area (Figure 2). For example, specimen CHN0610H from a patient in Yongzhou in Hunan Province and specimen CHN0633D from a local dog were in subgroup IIa, and human specimen CGZ0518H from Qianxinan in Guizhou Province were in group III, which includes specimen CGZ0505D from a dog in the same city. However, specimen CHN0635H from a patient in Hunan Province is more closely related to specimens from dogs in Guangxi Province (e.g., CGX0601D).

**Figure 3:**
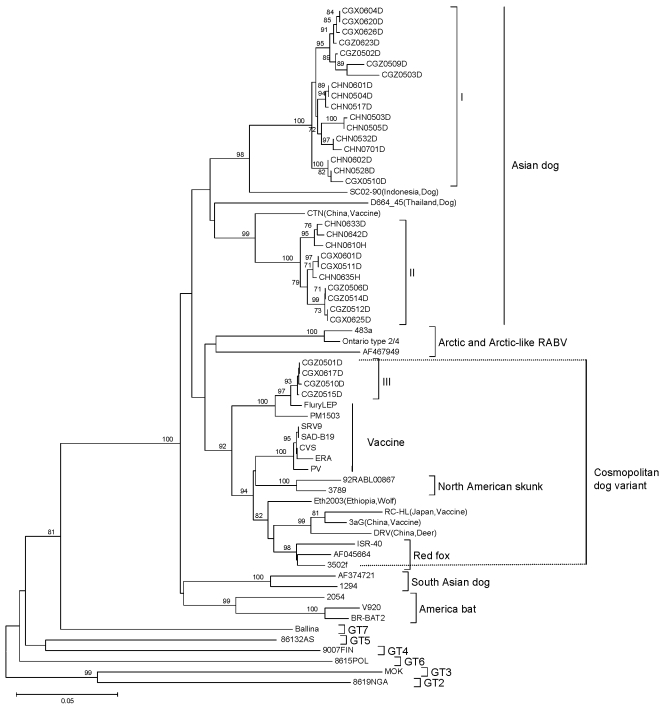
Neighbor-joining phylogenetic tree of 60 specimens of rabies virus (RABV) from the People’s Republic of China, 2005–2007, and different genotypes (GTs) from other areas based on a 720-nt (nt 704–nt 1423) nucleoprotein (N) gene fragment of RABV. Numbers at each node indicate degree of bootstrap support; only those with support >70% are indicated. Scale bar indicates nucleotide substitutions per site.

Most lineages and sublineages in the 60 samples from southern China were 100% identical (Figure 2). Thus, we chose representative samples to conduct a comparative phylogenetic analysis with RABV sequences from other countries and reservoir hosts (Figure 3). All virus specimens from southern China clustered in genotype 1 (GT1) and constituted a major branch (Asian dog; Figure 3) that is clearly segregated from other lyssavirus genotypes (GTs 2–7).

Most of the lineages in the samples from China clustered with other dog-related RABV lineages from Asia, such as groups I and II, which are closely related to samples from Indonesia and Thailand, respectively. There was no close relationship between RABV from China and RABV from other countries in southern Asia, such as India and Sri Lanka.

Some RABVs from Guizhou and Guangxi provinces (group III; Figure 3) were related to cosmopolitan dog-related RABV variants, including nearly all vaccine strains and variants associated with skunks, wolves, and red foxes. In addition, group III showed a distant relationship with Arctic and Arctic-like RABVs. All virus specimens from China were phylogenetically distant from bat-associated variants and lineages from the United States, Mexico, and Brazil.

RABV vaccine strains isolated at different times in China showed close links with at least 2 of 4 groups. Vaccine strain CTN isolated in Shandong Province (in eastern China) in the 1950s may have evolved with group II, whereas strain 3aG, isolated in Beijing in the 1930s, and deer strain DRV, isolated in Jilin Province (in northeastern China), are closely related to group III (Figure 3).

## Discussion

Guizhou, Guangxi, and Hunan provinces in China have reported the highest incidence of human rabies in recent years and were selected for national epidemic surveillance in 2005. According to national data on rabies surveillance, domestic dogs are the major infection source for humans and other domestic animals (*3*).

Phylogenetic analysis (Figure 2) showed that human RABV specimens generally belong to the same branch as dog RABVs from the same region, as exemplified by specimens HN0633D and HN0610H (subgroup IIa) and GZ0518H and GZ0505D (group III). This finding provides virologic evidence that domestic dogs are major reservoirs of human rabies epidemics (*2*). In addition, these results support the inference that RABVs from the same regions tend to group together and represent the same rabies focus or same geographically circumscribed outbreak (*20*–*22*).

We observed an infection rate of 2.3% in apparently healthy dogs from 15 cities in the 3 provinces. Previous surveys (*23*,*24*) in regions of high incidence of rabies showed different rates, ranging from 3.9% to 17.9% in dogs. Discrepancies in infection rates may be related to the sampling method (including nonclinically suspicious rabid dogs), different sample sizes, detection methods, and particular local variations. Regardless of the infection rate, the data indicate that rabies in dogs is prevalent in these regions and represents a cause of the high incidence of rabies in humans.

Phylogenetic analysis of specimens collected in the 3 provinces clearly shows that RABVs have a distinctive geographic correlation, in which group I was found predominantly in Hunan Province, group II in Guangxi Province, and group III in Guizhou Province (Figure 2). From an epidemiologic perspective, these groups may be interpreted as ongoing independent foci of dog rabies (or outbreaks).

Identity levels of deduced amino acid sequences of the 60 specimens ranged from 95.4% to 99.6%, which are higher than similarity values for corresponding nucleotide sequences. This finding indicates that most nucleotide variations in N gene coding sequence were synonymous mutations compatible with ongoing natural selection. Samples from different provinces had identical nucleotide sequences, which indicated that these viruses may have originated from the same outbreak or that a rabies focus had been spread between provinces (*13*,*14*).

Members of groups I and II were found in all 3 provinces selected for this study (Figure 2), which indicates that they have been spread throughout the region over time. The fact that subgroups Ic and IIa are integrated with virus samples from provinces distinct from their actual representative groups suggests that RABV translocation events are occurring between provinces (*25*). Phylogenetic inferences and epidemiologic and historical data suggest that ongoing rabies foci in Guangxi and Guizhou provinces, represented by subgroup Ic, had their origins in Hunan Province. Statistically supported independence of subgroup Ic within group I also suggests that such translocation events may have occurred in an isolated manner and may implicate dog RABV no longer circulating in Hunan Province. Similarly, subgroup IIa represents a rabies focus that had its origins in Guangxi Province and was then translocated to Hunan Province. Recent translocations from Hunan Province to Guangxi Province are represented by taxa CGX0612D, CGX0607D, CGX0510D, CGX0606D, and CGX0619D. Recent translocations from Guangxi Province to Hunan and Guizhou provinces represented by taxa CHNO635H, CGZ0514D, CGZ0506D, CGZ0512D, and CGZ0516D (Figure 2). Our results show trends in virus spread from current and historical occurrences in neighboring provinces.

Transprovincial spread of RABVs may be one of the forces responsible for exacerbating the dog rabies epizootic, as reflected by a human rabies epidemic. Specimen CGX0617D from Liuzhou in Guangxi Province (group III; Figure 2) is 100% identical to specimens from Guizhou Province, a finding that supports the suggestion that RABV was introduced into Liuzhou from Guizhou Province during the human rabies outbreak in 2004. Our findings are supported by previous studies in Guangxi Province (*26*,*27*), which suggested that different RABV lineages cocirculating in this province may correlate with an increased number of human rabies cases during 2003–2004 in Guangxi Province.

We sought to determine how transprovincial spread of RABVs occurred. The geographic nature of these rabies foci or groups suggests that dogs are not moving, per se, but that human-related activities may account for these phenomena. Persons in southern China are accustomed to eating dog meat because they believe it helps them resist dampness and cold in winter. In addition, many restaurants in cities and regions of southern China sell dog meat, which increases the demand for this type of meat. In rural areas of southern China, nearly every family owns several dogs, most of which are free-roaming, without special diets, and unvaccinated against rabies (to save costs). Sale of these dogs to restaurants can increase a farmer’s income (average 12–15 US dollars/dog). Free-roaming and unvaccinated dog populations may increase the likelihood of transprovincial spread of RABVs. Other human-related activities, such as persons migrating with their dogs may also contribute to long-distance spread of rabies. Dogs purchased by restaurants are soon killed for consumption. With the exception of butchers, there would be insufficient time to transmit RABV to other dogs and humans. Until now, persons in China who eat dog meat have not been not considered at risk for rabies because no related infections have been reported.

Specimens belonging to groups I and II, which are the predominant RABVs distributed across the 3 provinces, the Chinese vaccine strain CTN and strains from Southeast Asia form an isolated phylogenetic cluster. This finding demonstrates that strains from China and Southeast Asia may have the same origin (*11*,*14*,*28*–*30*). Southwestern China borders Vietnam, Laos, and Myanmar, countries into which persons in China have migrated over the past 2,000 years. Thus, dissemination of RABV has been historically linked across Southeast Asia.

Conversely, natural barriers such as the Himalayas and large rivers may have prevented RABV dissemination from the Indian subcontinent and central Asia into China (*20*). Our data indicate that RABV from these regions has not been recently transmitted into China. Enzootic dog RABV lineages from these regions (Indian dog and Arctic-like RABVs) are phylogenetically distinct from lineages in China, which supports this concept (*31*,*32*).

Groups I and II appear to have evolved from a common, long-term dog rabies enzootic in China. Group III appears to be more closely associated with widely spread dog-related RABVs represented by vaccine strains and other lineages established in wildlife in other regions (Figure 3). These results suggest 2 origins for rabies in dogs in the 3 provinces in southern China: an autochthonous origin and a more recent origin by introduction of a cosmopolitan dog RABV variant into China.

Approximately 57 years ago, Johnson (*33*) speculated that RABV strains from Europe were transmitted into China through Hong Kong and Shanghai. The attenuated 3aG strain, which was isolated in Beijing in 1931, and the DRV strain, which was isolated in Jilin Province in 2002, are closely related to group III. This finding implies that a group of viruses that originated in Europe is present in China and is still circulating. The hosts of this group include not only domestic dogs but also other mammals likely infected by rabid dogs (DRV strain). Alternatively, the similarity among some RABVs circulating in dogs in China and international vaccine strains (*34*) should motivate health authorities in China to revisit quality standards and adequacy for use of attenuated rabies vaccines to ensure that vaccine-related cases do not occur.

In conclusion, spread of RABVs from high-incidence regions, particularly by the long-distance migration or transprovincial movement of dogs caused by human-related activities, may be one of the causes of recent massive human rabies epidemics in southern China. We identified only 1 rabies case in a deer in China; it had evidence of spillover infection from a rabid dog. Although no evidence was found of independent rabies enzootics sustained by wildlife in China, multidisciplinary studies are required to improve rabies surveillance to identify potential alternative sources of human infection. Creation of a sensitive laboratory-based surveillance system for animal rabies detection is a prerequisite for enabling effective dog vaccination campaigns. Integration of enhanced surveillance, molecular analysis, local canine population management, and efficient rabies vaccination coverage in dogs are essential to prevent rabies from being transmitted by rabid dogs to humans.
